# Lifetime Body Mass Index Trajectories and Contrasting Lung Function Abnormalities in Mid‐Adulthood: Data From the Tasmanian Longitudinal Health Study

**DOI:** 10.1111/resp.14882

**Published:** 2025-01-26

**Authors:** Gulshan B. Ali, Adrian J. Lowe, E. Haydn Walters, Jennifer L. Perret, Bircan Erbas, Caroline J. Lodge, Gayan Bowatte, Paul S. Thomas, Garun S. Hamilton, Bruce R. Thompson, David P. Johns, John L. Hopper, Michael J. Abramson, Dinh S. Bui, Shyamali C. Dharmage

**Affiliations:** ^1^ Centre for Epidemiology and Biostatistics, Melbourne School of Population and Global Health University of Melbourne Melbourne Victoria Australia; ^2^ Murdoch Children's Research Institute, Royal Children's Hospital Melbourne Victoria Australia; ^3^ School of Medicine University of Tasmania Hobart Tasmania Australia; ^4^ School of Psychology and Public Health La Trobe University Victoria Australia; ^5^ Prince of Wales Clinical School, Faculty of Medicine UNSW New South Wales Australia; ^6^ Monash Lung, Sleep, Allergy and Immunology Monash Health Victoria Australia; ^7^ School of Clinical Sciences Monash University Victoria Australia; ^8^ Melbourne School of Health Science The University of Melbourne Melbourne Victoria Australia; ^9^ School of Public Health & Preventive Medicine Monash University Melbourne Victoria Australia

**Keywords:** body mass index trajectories, COPD, lifetime growth patterns, lifetime obesity, lung function decline, obesity trajectories, obstructive‐lung function, poor lung function, restrictive‐lung function

## Abstract

**Background and Objective:**

The impact of lifetime body mass index (BMI) trajectories on adult lung function abnormalities has not been investigated previously. We investigated associations of BMI trajectories from childhood to mid‐adulthood with lung function deficits and COPD in mid‐adulthood.

**Methods:**

Five BMI trajectories (*n* = 4194) from age 5 to 43 were identified in the Tasmanian Longitudinal Health Study. Lung function outcomes were defined using spirometry at 45 and 53 years. Associations between these BMI trajectories and lung function outcomes were investigated using multivariable regression.

**Results:**

Compared to the average BMI trajectory, the child's average‐increasing BMI trajectory was associated with greater FVC decline from 45 to 53 years (β = −178 mL; 95% CI −300.6, −55.4), lower FRC, ERV and higher TLco at 45 years, lower FVC (−227 mL; −345.3, −109.1) and higher TLco at 53 years. The High BMI trajectory was also associated with lower FRC, ERV and higher TLco at 45 years, while spirometric restriction (OR = 6.9; 2.3, 21.1) and higher TLco at 53 years. The low BMI trajectory was associated with an obstructive picture: lower FEV_1_ (−124 mL; −196.4, −51.4) and FVC (−91 mL; −173.4, −7.7), and FEV_1_/FVC (−1.2%; −2.2, −0.1) and higher ERV and lower TLco at 45 and 53 years. A similar pattern was found at 53 years. No associations were observed with spirometrically defined COPD.

**Conclusion:**

Our findings revealed contrasting lung function abnormalities were associated with high, subsequently increasing, and low BMI trajectories. These results emphasise the importance of tracking changes in BMI over time and the need to maintain an average BMI trajectory (BMI‐Z‐score 0 at each time point) throughout life.

## Abstract 2

Persistently high and child average increasing BMI trajectories from early childhood to mid‐adulthood, were at higher risk of lung function decline and spirometric restriction in mid‐adulthood, while being in the persistently low BMI trajectory was associated with spirometric obstruction.

**Summary**

- Persistently high and child average increasing BMI trajectories from early childhood to mid‐adulthood, were at higher risk of lung function decline and spirometric restriction in mid‐adulthood, while being in the persistently low BMI trajectory was associated with spirometric obstruction.

**Abbreviations**
ATS: American Thoracic Society
BIC: Bayesian information criterion
BMI: body mass index
COPD: chronic obstructive pulmonary disease
DAG: directed acyclic graph
ERS: European Respiratory Society
FEV1: forced expiratory volume in one second
FRC: functional residual capacity
FVC: forced vital capacity
GBTM: group‐based trajectory modelling
GLI: global lung function initiative
LLN: lower limit of normal
Post‐BD: post bronchodilator
SGA: small for gestational age
TAHS: Tasmanian Longitudinal Health Study
TLC: total lung capacity
TLco: transfer factor for carbon monoxide

## 1 Introduction

An accelerated decline in lung function, leading to adverse outcomes such as the potential development of chronic obstructive pulmonary disease (COPD) in adulthood, has been linked to premature mortality and other non‐communicable diseases, including cardiovascular disease and diabetes [1, 2, 3]. COPD is currently the third leading cause of death, accounting for 3.3 million deaths annually, imposing a significant burden on healthcare systems [4].

This analysis particularly focuses on the role of lifetime body mass index (BMI) change as a modifiable risk factor for lung function deficits and COPD in mid‐adulthood [5, 6]. Several studies have investigated the relationship between BMI and lung function abnormalities; however, their results have been inconsistent. Some studies reported an association between low BMI and spirometric restriction [7, 8]. Conversely, others have suggested that a high BMI might increase the risk of adult spirometric restrictive patterns [9]. A potential source of these inconsistencies may be the reliance on BMI measurement taken at a single point in time, which may fail to capture variations or dynamic changes in adiposity over the life course.

In contrast, repeated measures of lifetime BMI at different time points can offer valuable insights into the variability, age of onset, duration and intensity of adiposity over time [10]. This approach enables differentiation of the effects of distinct BMI patterns or ‘trajectories’ on lung function outcomes in mid‐adulthood [11]. Therefore, we hypothesized that statistically modelling changes in adiposity throughout the life course might identify trajectories associated with varying risks and provide crucial insights into critical exposure windows.

Several studies have documented that different published patterns of lifetime BMI trajectories are differentially associated with various chronic health conditions, with individuals following high BMI trajectories being at heightened risks of developing multiple non‐communicable diseases [12, 13, 14, 15]. However, no studies have explored the relationship of lifetime BMI trajectories with lung function deficits and COPD in middle age. Moreover, our previous work has indicated that both Child Average‐Increasing and consistently high BMI trajectories from childhood to middle age were associated with new‐onset asthma in middle‐age [16].

Given this gap in existing knowledge, we investigated the association between BMI trajectories from childhood to middle age (from 5 to 43 years) and various lung function parameters at 45 and 53 years, including spirometry, change in spirometry, total lung capacity (TLC), functional residual capacity (FRC), expiratory reserve volume (ERV), transfer factor for carbon monoxide (TLco) and spirometrically defined COPD.

## 2 Methods

### 2.1 Study Population and Design

The current study used data from the Tasmanian Longitudinal Health Study (TAHS), a prospective cohort study. The methodology was extensively outlined and published previously [17]. At baseline, the children (7 years old) underwent spirometry, and their parents completed a detailed questionnaire on various respiratory outcomes. Follow‐up assessments were performed at ages 13, 18, 30, 45, 50 and 53, with surveys completed and spirometry measured following the ATS and ERS joint guidelines [18]. Post‐BD spirometry and TLco were measured at ages 45 and 53 years, while static lung volumes were measured at 45 years (see in the Supporting Information repository method 1).

### 2.2 Exposure: Body Mass Index Trajectories

We have previously published a detailed procedure for developing BMI trajectories. BMI was calculated at eight‐time points using group‐based trajectory modelling (GBTM) on 4194 participants with at least 3‐time points [19].

### 2.3 Outcomes: Lung Function

Lung function outcomes were analysed using four outcome variables.

- *Spirometry*

Spirometry indices as a continuous variable at ages 45 and 53 were forced expiratory volume in the first second (FEV_1_), forced vital capacity (FVC) and the ratio of the FEV_1_ to FVC (FEV_1_/FVC).

- *Change in spirometry*

Change in spirometry was calculated by subtracting lung function indices (FEV_1_, FVC and FEV_1_/FVC) at 45 from lung function indices at 53 years and adjusted for baseline lung function.

- *Spirometry phenotypes*

GLI reference values were used to derive LLN (lower limit of normal) [20]. Spirometry phenotypes at 45 and 53 years were defined as a four‐level variable that was mutually exclusive:

1. Spirometric restriction‐only was defined as FVC < LLN and FEV_1_/FVC ratio ≥ LLN.
2. Obstructive‐only spirometry was defined as FVC ≥ LLN and the FEV_1_/FVC ratio < LLN.
3. Mixed pattern was defined as both FVC and the FEV_1_/FVC ratio < LLN.
4. Normal spirometry was defined as the FVC and FEV_1_/FVC ratio ≥ LLN [21].

### 2.4 Chronic Obstructive Pulmonary Disease

COPD at the age of 45 and 53 years was defined spirometrically as post‐BD FEV_1_/FVC ratio < LLN.

### 2.5 Statistical Analysis

The associations of BMI trajectories with continuous lung function parameters at 45 and 53 years and change in lung function from 45 to 53 years were evaluated with linear regression.

The association with COPD and spirometry phenotype at 45 and 53 years was evaluated using logistic and multinominal regression respectively. Initially, the spirometric phenotype was defined as a four‐level variable, but the mixed group was excluded as there were only six participants at age 45 and 7 at age 53 in this group. We then compared spirometric restriction and obstructive only versus normal for this variable.

All associations were adjusted for the potential confounders selected by developing a directed acyclic graph (DAG) based on a literature review. Smoking was considered an effect modifier, as the information was available at 43 and 53 years. (see Figures S1 and S2 in the Supporting Information) [22] (see Supporting Information repository methods 2 and 3 for interaction and confounding).

We performed an additional regression analysis of BMI trajectories with static lung volumes (TLC, FRC and ERV) and TLco to support the spirometry findings in a subset for these parameters at 45 and 53 years (Supporting Information repository method 4).

## 3 Results

### 3.1 Early Life Characteristics of BMI Trajectories

**FIGURE 1:**
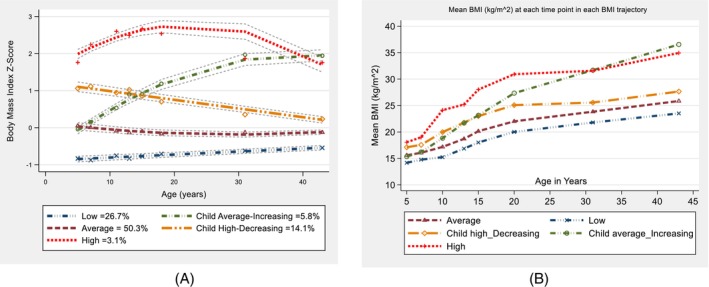
(a, b) Body mass index (Z‐score and mean BMI) trajectory classes across ages from 5 to 43 years.

Five distinct BMI trajectories were identified from 5 to 43 years of age for 4194 participants: Average, low, high, child high‐decreasing and child average‐increasing (Figure 1) [16]. Illustration of the BMI trajectories can be found in the Supporting Information. The distributions of sex, childhood asthma and bronchitis were similar across all five trajectories (Table 1). Childhood %predicted FEV_1_ and FVC were lower in the Low BMI trajectory, with the prevalence of having been pre‐term or small for gestational age (SGA) being higher in this group. Furthermore, the proportions of participants with low birth weight, parental smoking and maternal asthma were higher in the child average‐increasing and high BMI trajectories. In addition, the proportion of parents who worked as labourers was higher in child average‐increasing and high BMI trajectories.

**TABLE 1:** Early life characteristics of the BMI trajectory groups.

Characteristics	Average (*N* = 2109)	Low (*N* = 1120)	Child high‐decreasing (*N* = 592)	Child average‐increasing (*N* = 245)	High (*N* = 128)
Early life characteristics					
Sex—male—% (*n*)	52.8 (1114)	50.0 (560)	52.7 (312)	42.9 (105)	50.0 (64)
%Predicted FEV_1_ at 7 years M (SD)	99.3 (12.4)	97.2 (11.7)	101.1 (13.7)	99.1 (12.7)	102.1 (12.1)
%Predicted FVC at 7 years M (SD)	97.6 (11.9)	94.8 (11.6)	100.0 (12.3)	97.4 (12.5)	100.6 (11.6)
Height (m) at 7 years M (SD)	1.20 (0.05)	1.19 (0.05)	1.22 (0.05)	1.20 (0.05)	1.22 (0.05)
Pre‐term—% (*n*)	11.4 (119)	15.9 (94)	15.3 (42)	5.8 (7)	9.1 (6)
Low birthweight—% (*n*)	5.6 (66)	8.6 (58)	2.2 (7)	9.8 (14)	2.7 (2)
Small for gestation—% (*n*)^a^	20.5 (189)	27.8 (147)	12.2 (29)	24.5 (27)	24.1 (14)
Childhood asthma—% (*n*)	17.5 (364)	15.5 (171)	18.8 (111)	19.9 (46)	14.8 (19)
Childhood bronchitis—% (*n*)	50.9 (1060)	47.5 (524)	46.1 (271)	53.1 (129)	52.3 (67)
Social class—% (*n*)					
Managers/professional	22.1 (447)	21.5 (230)	25.9 (147)	16.4 (38)	16.8 (20)
Tradespersons/Adv‐clerical	6.8 (138)	6.6 (71)	5.8 (33)	6.1 (14)	6.7 (8)
Inter‐med‐clerical/production	30.5 (617)	30.5 (327)	26.9 (152)	30.2 (70)	26.1 (31)
Elementary clerical	28.7 (579)	27.8 (297)	27.2 (154)	29.7 (69)	29.4 (35)
Labourers/rel‐workers	11.8 (239)	13.5 (144)	14.1 (80)	17.6 (41)	21.0 (25)
Mother's smoking—% (*n*)	33.9 (680)	31.1 (332)	34.0 (193)	35.8 (83)	48.4 (60)
Father's smoking—% (*n*)	56.4 (1109)	59.5 (622)	59.8 (331)	62.2 (140)	62.8 (76)
Mother's asthma—% (*n*)	10.9 (219)	9.9 (106)	11.1 (63)	15.9 (37)	12.9 (16)
Father's asthma—% (*n*)	11.3 (222)	9.5 (99)	10.8 (60)	10.8 (24)	9.8 (12)

*Note*: Out of the total sample (*n* = 4194). For continuous variables, we used ANOVA, and for categorical variables, the *χ* ^2^ test. Some data presented here are republished from Ali et al. ERJ 2022 [16] with permission.

^a^ Gestational age was known for 2100 participants, birthweight for 2391 participants, and small for gestational age for 1855 participants (406 were small for gestational age).

### 3.2 Associations Between BMI Trajectories, Spirometry, Phenotypes and COPD

#### 3.2.1 Low BMI Trajectory

**FIGURE 2:**
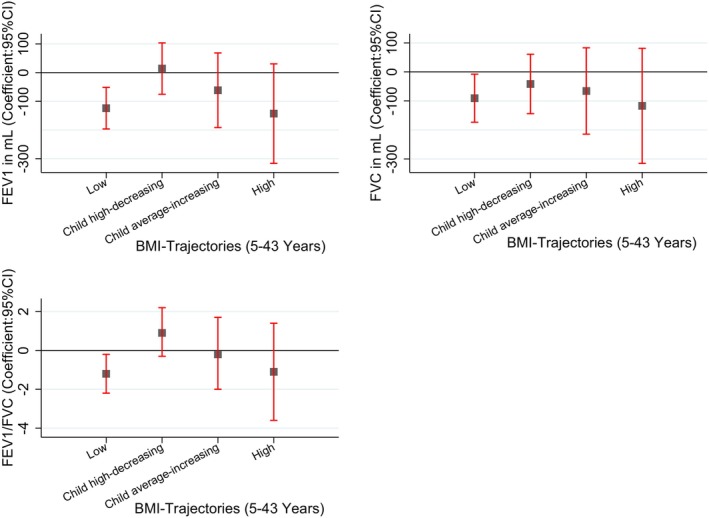
Association of BMI trajectories with the lung function parameters at 45 years. **Finding**: Compared to the average trajectory, low BMI trajectory was associated with low FEV, FVC, and ratio. **Adjusted**: Sex, type of feeding in the first 3 months, no of siblings, chest illness, tonsillectomy, pneumonia, childhood food allergy, bronchitis, social class during childhood, mother's employment, mother's age, mother's asthma, mother's smoking, father's asthma, father's smoking, adulthood education, adulthood food allergy, height at 45 years, and age at 45 years.

**FIGURE 3:**
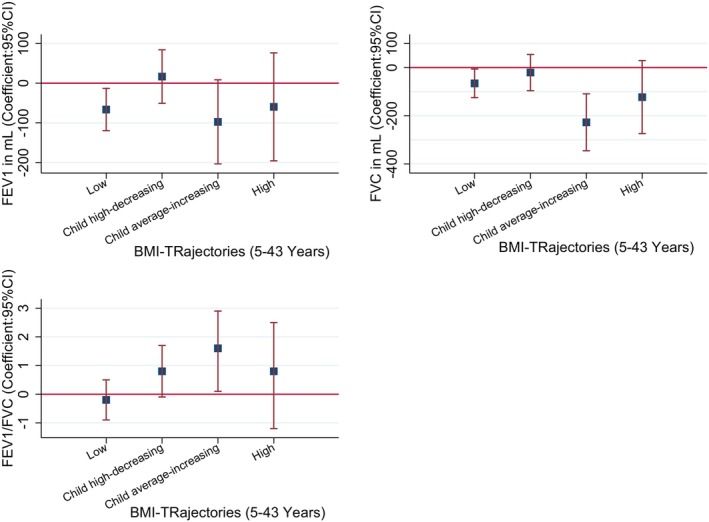
Association of BMI trajectories with the lung function parameters at 53 years. **Finding**: Compared to the average trajectory, low BMI trajectory was associated with low FEV_1_ and FVC, while child average‐increasing BMI trajectory was associated with low FVC. **Adjusted**: sex, type of feeding in the first 3 months, no of siblings, chest illness, tonsillectomy, pneumonia, childhood food allergy, bronchitis, social class during childhood, mother's employment, mother's age, mother's asthma, mother's smoking, father's asthma, father's smoking, adulthood education, adulthood food allergy, height at 53, and age at 53 years.

**FIGURE 4:**
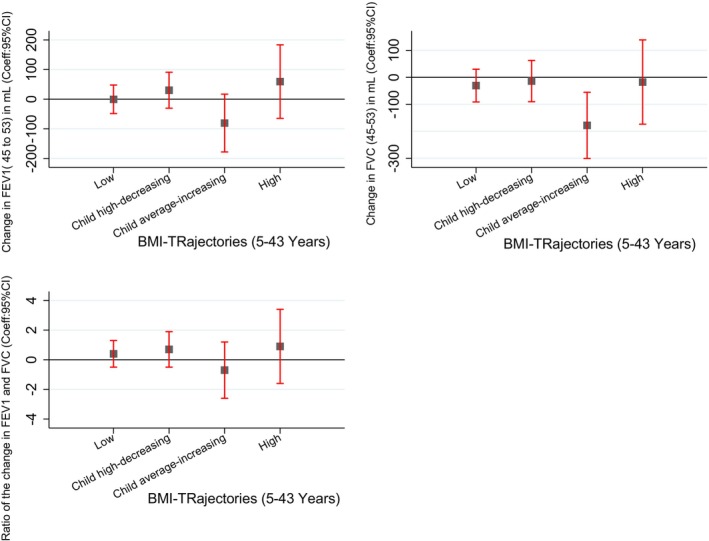
Association of BMI trajectories with the lung function decline from 45 to 53 years. **Finding**: Compared to the average trajectory, child average‐increasing BMI trajectory was associated with greater FVC decline. **Adjusted**: Sex, type of feeding in the first 3 months, no of siblings, chest illness, tonsillectomy, pneumonia, childhood food allergy, bronchitis, social class during childhood, mother's employment, mother's age, mother's asthma, mother's smoking, father's asthma, father's smoking, adulthood education, adulthood food allergy, current employment, baseline lung function, change in height 45–53 and change in age 45–53.

Compared to the Average BMI trajectory, the Low BMI trajectory was associated with lower FEV_1_, FVC and FEV_1_/FVC ratio at age 45 (Figure 2, Table S1 in the Supporting Information). Similarly, at 53 years, the low BMI trajectory was associated with lower FEV_1_ and lower FVC (Figure 3, Table S2 in the Supporting Information). This trajectory was not associated with the change in lung function from 45 to 53 years (Figure 4, Table S3 in the Supporting Information). No associations were found with spirometry phenotypes or spirometrically defined COPD at 45 (Figures S3 and S4, Tables S4 and S5 in the Supporting Information) or 53 years (Figures S5 and S6, Tables S6 and S7 in the Supporting Information).

#### 3.2.2 Child High‐Decreasing BMI Trajectory

The Child High‐decreasing trajectory was not associated with any spirometry measures (Figures 2, 3, 4, Tables S1–S3 in the Supporting Information), spirometry phenotypes or COPD at 45 (Figures S3 and S4, Tables S4 and S5 in the Supporting Information) or 53 years (Figures S5 and S6, Tables S6 and S7 in the Supporting Information).

#### 3.2.3 Child Average‐Increasing BMI Trajectory

The child average‐increasing BMI trajectory was not associated with spirometry measures at 45 years (Figure 2, Table S1 in the Supporting Information). However, at 53 years, it was associated with lower FVC and higher FEV_1_/FVC ratio (Figure 3, Table S2 in the Supporting Information). It was also associated with a significant decline in FVC from 45 to 53 years (Figure 4, Table S3 in the Supporting Information). This BMI trajectory was not associated with spirometry phenotypes or COPD at either 45 (Figures S3 and S4, Tables S4 and S5 in the Supporting Information) or 53 years (Figures S5 and S6, Tables S6 and S7 in the Supporting Information).

#### 3.2.4 High BMI Trajectory

We did not see any evidence of an association between the High BMI trajectory and spirometry measures at age 45 or 53, nor change in spirometry from 45 to 53 years (Figures 2, 3, 4, Tables S1–S3 in the Supporting Information); however, a trend can be observed for both lower FEV_1_ and FVC.

This BMI trajectory did not show any association with spirometry phenotypes or COPD at 45 years (Figures S3 and S4, Tables S4 and S5 in the Supporting Information). However, at age 53 years, there was a significant association with a spirometric restriction phenotype (Figure S5 and Table S6 in the Supporting Information) but not with COPD (Figure S6 and Table S7 in the Supporting Information).

#### 3.2.5 Interactions

We did not see any interactions with sex, smoking, BMI or childhood lung function (Table S8 in the Supporting Information). However, we observed interactions between BMI trajectories and childhood asthma (p‐interaction: FEV_1_ = 0.04 and FVC = 0.07) on spirometry at 53 years. The association between the low BMI trajectory and lower FEV_1_ and lower FVC was more pronounced in those with childhood asthma (Table S9 in the Supporting Information).

#### 3.2.6 Complex Lung Function Outcome

**FIGURE 5:**
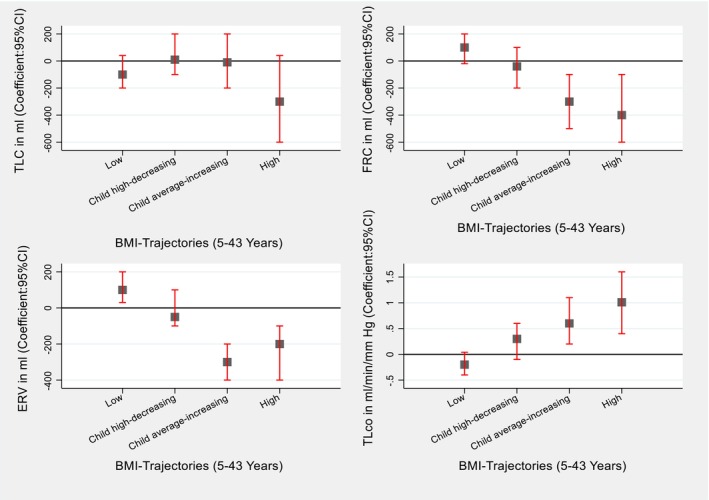
Association of BMI trajectories (5–43 years) with the TLco and Static Lung Volumes at 45 years. **Finding**: Compared to the average trajectory, high and child average‐increasing BMI trajectories were associated with low FRC and ERV and high TLco. **Adjusted**: sex, type of feeding in the first 3 months, chest illness, tonsillectomy, pneumonia, childhood food allergy, bronchitis, social class during childhood, mother's employment, mother's age, mother's asthma, mother's smoking, father's asthma, father's smoking, adulthood education, adulthood food allergy height at 45 years and age at 45 year.

**FIGURE 6:**
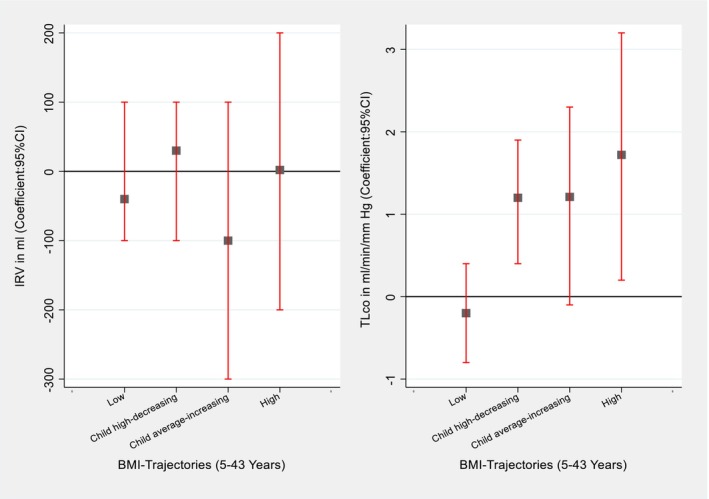
Association of BMI trajectories (5–43 years) with the TLco and IRV at 53 year. **Finding**: Compared to the average trajectory, high BMI trajectory was associated with high TLco. **Adjusted**: Sex, type of feeding in the first 3 months, chest illness, tonsillectomy, pneumonia, childhood food allergy, bronchitis, social class during childhood, mother's employment, mother's age, mother's asthma, mother's smoking, father's asthma, father's smoking, adulthood education, adulthood food allergy, height at 53 years and age at 53 years.

For the additional analysis with static lung volumes, we observed a link between the child average‐increasing and high BMI trajectories with decreased static lung volumes (TLC, FRC and ERV) and increased TLco at 45 and 53 years. We also observed an increased ERV and a slightly decreased TLco with the low BMI trajectory (Figures 5 and 6, Tables S10 and S11 in the Supporting Information).

An overall summary of these findings can be found in Table 2.

**TABLE 2:** Summary of the findings of the associations of BMI trajectories with lung function at 45 years, 53 years and the change from 45 to 53 years.

BMI trajectories	Average	Low	Child high‐decreasing	Child average‐increasing	High
Lung function indicators at 45 years					
FEV_1_	—	↓			
FVC	—	↓			
FEV_1_/FVC	—	↓			
Obstruction only	—	~↑			
Spirometric restriction only	—				
COPD	—				
TLC	—				~↓
FRC	—			↓	↓
RV	—				
ERV		↑		↓	↓
TLco	—	~↓	~↑	↑	↑
Lung function indicators at 53 years					
FEV_1_	—	↓		~↓	
FVC	—	↓		↓	
FEV_1_/FVC	—		~↑	↑	
Obstruction only	—				
Spirometric restriction only	—				↑
COPD	—				
IRV	—				
TLCO	—		↑	~↑	↑
Lung function declines from 45 to 53 years					
FEV_1_	—				
FVC	—			↓	
FEV1/FVC	—				

*Note*: ↓&↑: The direction of the significant associations (lower or higher than average trajectory with a *p*‐value: < 0.05). ~: Moderately significant (*p*‐value: 0.05 < 0.1). ↓: For lung function decline, this sign indicates greater decline.

Abbreviations: COPD, chronic obstructive pulmonary disease; TLC, total lung capacity; FRC, functional residual capacity; ERV, expiratory reserve volume; TLco, transfer factor for carbon monoxide.

## 4 Discussion

Using distinct BMI trajectories from 5 to 43 years of age in a well‐characterised respiratory cohort, we observed that compared to the average trajectory, individuals belonging to the child average‐increasing trajectory had a more rapid decline in FVC from 45 to 53 years and lower FVC, and higher FEV_1_/FVC at 53 years, suggesting an overall spirometric restrictive pattern. Participants belonging to the High trajectory also had an increased risk of spirometric restriction at the age of 53 years. Additionally, these trajectories were associated with reduced static lung volumes and increased TLco, confirming that they were associated with extra‐pulmonary restrictive deficits. Interestingly, belonging to the low trajectory was associated with lower FEV_1_, FVC and FEV_1_/FVC ratio at 45 years and lower FEV_1_ and FVC at 53 years, with a more substantial effect on FEV_1_, thus showing a dominant obstructive lung function pattern. Although the (relatively) Low BMI trajectory remained in the technically normal BMI range (BMI Z score between −1 and 0 SD), its lung function pattern in fact resembled the obstructive picture commonly seen with extremely underweight individuals [7]. No associations were observed between BMI trajectories and spirometrically defined COPD in middle age, but this may be due to limited power.

Due to a lack of suitable long‐term prospective studies, the relationship between lifetime BMI patterns and adult lung function has received little attention in research. Nonetheless, some evidence suggests that persistently High BMI groups during early childhood were associated with low FEV_1_/FVC ratio in children and adolescents [23, 24]. We have previously found infants belonging to persistently High BMI trajectories from 0 to 2 years showed lower FEV_1_/FVC ratio and FEV_1_. In contrast, the low BMI trajectory was associated with a low FEV_1_ and FVC [25]. A recent ECRHS study revealed that rapid weight gain among normal‐weight individuals over time was tied to a sharp decline in lung function [26]. Peralta et al. estimated the change in BMI from 20 to 40 years, reporting that baseline normal weight individuals with significant weight gain (≥ 1 kg per year) during follow‐up had lower FVC during the same period compared to average weight gain, despite having similar FVC levels at baseline. Similarly, in a Swedish population, increased BMI during adult life was reported to be associated with faster FVC decline [27]. However, these studies did not consider BMI changes from early life to middle age, a critical aspect of our investigation. These previous studies observed parallel weight and lung function changes over time; in contrast, we investigated the lung function change later to avoid reverse causation. Also, our study indicated that the intensity and duration of high BMI, that is, in the persistently high trajectory showed greater restriction compared to the child average increasing trajectory, which was novel, not having been shown in previous studies using a BMI at a single time point [7, 8, 9]. Our study offered evidence that both high and low BMI trajectories from early to adult life could impact subsequent lung function patterns differently, especially in terms of restrictive vs. obstructive physiological patterns. This has been demonstrated in previous studies, but our findings highlight the importance of considering long‐term BMI trajectories rather than single‐point measures.

The participants belonging to the child average‐increasing BMI trajectory showed an increase in BMI during adult life, associated with an accelerated decline in FVC from 45 to 53 years, with a spirometric restrictive pattern evident by 53 years compared to the Average trajectory. This group displayed nearly twice the rate of FVC decline over the latter 8 years compared to the Average BMI trajectory. This aligns with a recent American study that reported that more rapid increases in BMI were associated with greater rates of decline in both FEV_1_ and FVC [28]. The High BMI trajectory manifested extra‐pulmonary lung restriction at 53 years, supporting the evidence that persistent high BMI contributes to spirometric restriction [29]. Interestingly, the child average‐increasing and high BMI trajectories exhibited reduced lung volumes (ERV, FRC and TLC) and increased TLco, aligning with findings with spirometric lung function outcomes for these trajectories. Interestingly, the child average‐increasing trajectory presented a restrictive pattern across various lung function measures. This trajectory increases to mean BMI of 35 kg/m^2^ after 30 years of age. This relatively sharp increase would be likely due to a combination of lifestyle/environmental and genetic factors. In contrast, the high trajectory displayed significant associations solely with spirometric restriction. This distinction may be attributed to distinct pathophysiological mechanisms among those who consistently accumulate adiposity over time.

Mechanistically, the encasement of the thorax by excess fat or abdominal adiposity leading to diaphragmatic elevation results in the demonstrated spirometric restriction. It is also likely to result in elevated free fatty acids and systemic inflammation [30]. Numerous studies have indicated that systemic inflammation resulting from high levels of free fatty acids may lead to lung alveolar endothelial cell dysfunction [30, 31]. Endothelial dysfunction induced by systemic inflammation may lead to pulmonary vascular damage and lung tissue injury. However, the high lung transfer factor does not support such effects, which suggests the restriction is due to the mechanical impacts of extra‐pulmonary restriction. Our findings are consistent with other studies advocating rapid weight gain or persistently being overweight over time associated with poor lung function in mid‐adulthood. Given these findings, we suggest that the community should be encouraged to avoid excess weight gain throughout life, and from our data from early life to middle age, to promote lung health.

Conversely, the low BMI trajectory exhibited obstructive lung function at 45 and 53 years, consistent with findings from some other studies [32]. The Low trajectory group had an obstructive pattern at both time points and lower lung function at age 7, suggesting lung function impairment that commenced in early life. Although the BMI Z‐scores for this group were in the statistically normal Z‐score range, their lung function remained suboptimal and obstructed compared to the Average trajectory group. Poor lung function in this trajectory could be due to compromised lung/airway development during childhood or early life due to lifestyle/environmental insults and/or genetic predisposition insults. In addition, SGA and pre‐term birth were more prevalent in this trajectory compared to other trajectories, suggesting perhaps some element of broncho dysplasia, although the association was not significant. Individuals with a long‐term low BMI may have been more liable to childhood infections, which might also affect adult airways rather than whole lung development [33]. A weaker diaphragm and intercostal and abdominal muscle weakness could reduce respiratory muscle strength during early life [7, 34]. Notably, the adverse impact of belonging to the low trajectory was more pronounced in individuals with a history of childhood asthma, and perhaps this has given a legacy of airway dysfunction. Notably, lung volumes, particularly ERV, typically reduced in obesity, demonstrated an opposing trend within the low BMI trajectory group. Individuals in this group exhibited increased lung volumes (ERV and FRC), with a slight tendency toward a modest reduction in TLco at ages 45 and 53. This observation suggests the possibility of limited emphysema and airway damage in low trajectory, although statistical significance with TLco was not attained [35].

Our study has several strengths. First, we investigated these associations among a population‐representative sample with a relatively large cohort size, with good follow‐up extended over six decades. Thus, we have multiple anthropometric measurements from childhood to middle age, which provided an opportunity to investigate long‐term patterns of change in BMI from early childhood to middle age, thereby enabling a robust classification of BMI trajectories over this long period. Second, body weight and height were measured repeatedly from 5 to 20 years, capturing the BMI trajectory transition during the early rapid growth period. Third, BMI trajectories were fitted from childhood to 43 years of age, but outcomes were evaluated at both 45 and with change to 53 years to avoid reverse causation. Fourth, we used multiple outcome variables of lung function to confirm the associations. Finally, using objective GBTM was a strength, as it enabled us to identify patterns of BMI growth from early life to middle age.

However, this study also has several limitations. Weight and height were measured at ages 5, 7, 10, 13, 15 and 20, but self‐reported at 30 and 43 years. However, studies suggest that the discrepancy between actual and self‐reported weight has declined since the late 1980s [36]. To address this potential methodological issue of using different data sources, we assessed the correlation between self‐reported data at 43 years and the measured data at 45 years in another phase in a nested group (*n*—1360) was very strong (r = 0.88, *p* ≤ 0.001). Therefore, we believe that using two data sources at both time points did not influence our findings. While there was potential bias due to attrition, neither exposure nor lung function indices at earlier times were associated with attrition in our study (Table S12 and S13). In addition, the baseline characteristics of the participants were similar to those included in computed BMI trajectories and the remainder of the entire cohort. Therefore, it was unlikely that attrition bias substantively influenced these findings, hence they are generalizable to the population of Tasmania, Australia.

Another limitation of this study was that information on some of the important confounders, including maternal obesity, diet and physical activity, was not available. This may have led to some residual confounding in the observed associations. Additional adjustment for SGA in a small subset did not alter the pattern of the associations, and the magnitude of the effects was the same with or without adjustment. Hence, we assume SGA was not acting as a confounder in the subset where it was available. Additionally, no information on medication use was available for our study, which could confound the associations between BMI and lung function deficits. Future studies should consider medication data as part of the analysis to provide a more comprehensive understanding of potential confounders, as there is some evidence that inhaled steroids are associated with increased BMI [37].

A key limitation of this study was the use of BMI, which, although widely used, is an imperfect surrogate for adiposity. It does not capture fat distribution or muscle mass, which could influence lung function outcomes. Future studies should incorporate more precise body composition measurements, such as dual‐energy x‐ray absorptiometry (DEXA) or bioelectrical impedance analysis (BIA), to better understand how different body compartments influence lung function trajectories. Furthermore, the lack of association of these trajectories with COPD may be due to the limited number of COPD cases, as the numbers with such advanced airway obstruction in each trajectory were not high, with the disease not fully expressed at 45 or 53 years of age. Defined COPD using only spirometry is another limitation of this study, as we do not have CT imaging yet in the whole cohort.

In *conclusion*, extreme BMI trajectories over time are associated with lung function abnormalities in middle age. Those with an early average but increasing BMI over time were at a notably higher risk of accelerated FVC decline from middle age and spirometric restriction in later life. Those in the lifetime high BMI group were also at higher risk of spirometric restriction. In contrast, those in the persistently low BMI group were at higher risk of obstructive lung function patterns in middle age, likely due to persistent airway damage with the possibility of early emphysema.

Our findings have both clinical and public health implications. Visual presentation of BMI trajectories could educate people about the risks of being in the High BMI trajectory groups. It would be helpful to develop weight management programs for young adults to encourage behaviour change (diet and physical activity), focusing on strategic times and the intensity of BMI. The evidence from this study could help develop targeted preventive strategies for poor lung function in later life. Having an anthropometric growth record for the lifetime of individuals could be beneficial in assessing the population at risk and identifying the best time to intervene by modifying behaviours to lose weight. Given that the mechanisms to explain the link between BMI and lung function impairment remain uncertain, this study also provides an impetus for further investigation, focusing on possible underlying mechanisms that might help identify therapeutic targets.

## Author Contributions

**Gulshan B. Ali:** conceptualization (equal), data curation (lead), formal analysis (lead), investigation (lead), methodology (equal), software (lead), validation (equal), visualization (lead), writing – original draft (lead), writing – review and editing (lead). **Adrian J. Lowe:** conceptualization (equal), data curation (supporting), formal analysis (supporting), funding acquisition (supporting), investigation (supporting), methodology (supporting), supervision (equal), visualization (equal), writing – original draft (supporting), writing – review and editing (lead). **E. Haydn Walters:** funding acquisition (equal), investigation (supporting), writing – review and editing (equal). **Jennifer L. Perret:** investigation (supporting), writing – review and editing (equal). **Bircan Erbas:** investigation (supporting), writing – review and editing (equal). **Caroline J. Lodge:** investigation (supporting), writing – review and editing (equal). **Gayan Bowatte:** data curation (supporting), investigation (supporting), writing – review and editing (equal). **Paul S. Thomas:** investigation (supporting), writing – review and editing (equal). **Garun S. Hamilton:** investigation (supporting), writing – review and editing (equal). **Bruce R. Thompson:** investigation (supporting), writing – review and editing (equal). **David P. Johns:** investigation (supporting), writing – review and editing (equal). **John L. Hopper:** investigation (supporting), writing – review and editing (equal). **Michael J. Abramson:** funding acquisition (supporting), investigation (supporting), writing – review and editing (equal). **Dinh S. Bui:** conceptualization (equal), data curation (supporting), formal analysis (supporting), investigation (supporting), methodology (equal), supervision (equal), visualization (equal), writing – review and editing (lead). **Shyamali C. Dharmage:** conceptualization (lead), data curation (lead), formal analysis (supporting), funding acquisition (lead), investigation (lead), methodology (lead), resources (equal), supervision (lead), validation (supporting), visualization (supporting), writing – original draft (supporting), writing – review and editing (lead).

## Ethics Statement

The Tasmanian Longitudinal Health Study initial and follow‐up studies in 1968, 1974 and 1979 were approved by the Tasmanian Minister of Health and the human ethics review committee at the University of Tasmania. The 2002, 2010 and 2012 follow‐up studies were approved by the human ethics review committees at the University of Melbourne (approval number 040375), Tasmania (040375.1) and New South Wales (08094), the Alfred Hospital (1118/04) and Royal Brisbane and Women's Hospital health service district (2006/037) and conducted by the amended Declaration of Helsinki. Approval for the 53‐year follow‐up was obtained from the Human Research Ethics Committees at the University of Melbourne (reference no. 1238525), Tasmania (reference no. H0012710), New South Wales (reference no. HC12417), the Alfred Hospital (reference no. 367/12), Gold Coast Hospital and Health Service (reference no. HREC 12/QGC/175) and the Hunter New England Local Health District (reference no. HREC 12/HNE423). All participants provided written informed consent.

## Conflicts of Interest

Bircan Erbas and Paul S. Thomas were Editorial Board members of Respirology at the time of peer review and co‐authors of this article. They were excluded from all editorial decision‐making related to the acceptance of this article for publication. M.J.A. holds investigator‐initiated grants for separate research from Pfizer, Boehringer‐Ingelheim and GSK. He has undertaken an unrelated consultancy for Sanofi. He has also got a speaker's fee from GSK. S.C.D., A.J.L., J.L.P., M.J.A., C.J.L. and D.S.B. declare they have received research funds from GSK's competitively awarded Investigator Sponsored Studies program for unrelated research. S.C.D., A.J.L. and M.J.A. declare they have received funds from Sanofi for unrelated research. A.J.L. also declares he has received donations of interventional product (EpiCeram) from Primus Pharmaceuticals for unrelated research. The other authors included in this study declare that they have no competing interests. All authors declare no funding from any organisation or no financial relationship with any organisation that could appear to have influenced the submitted work.

## Supporting information


**Data S1.** Supporting Information.

## Data Availability

The data supporting this study's findings are available on request from the corresponding author. Due to privacy or ethics, the data are not publicly available.
