# Supplementary material for: Lifetime Body Mass Index Trajectories and Contrasting Lung Function Abnormalities in Mid‐Adulthood: Data From the Tasmanian Longitudinal Health Study
Source: Respirology. 2025 Jan 26;30(3):230–41. doi: 10.1111/resp.14882 (PMC11872284; doi:10.1111/resp.14882)
Supplement: Supplementary file 1 — Data S1. Supporting Information. [file RESP-30-230-s001.docx]

**SUPPORTING INFORMATION**

**Lifetime Body Mass Index Trajectories and Contrasting Lung Function Abnormalities in Mid-Adulthood, Data from the Tasmanian Longitudinal Health Study**

Gulshan B Ali1 MSc, Adrian J Lowe1,2 PhD, E Haydn Walters1,3 DM, DSc, Jennifer L Perret1 PhD, FRACP, Bircan Erbas4 PhD, Caroline Lodge1,2 PhD, Gayan Bowatte1 PhD, Paul S Thomas5 FRACP, Garun S Hamilton6,7 FRACP, PhD , Bruce R Thompson8, PhD, David Johns3 PhD, John Hopper1 PhD, Michael J Abramson9 PhD, FRACP, Dinh S Bui1 PhD *, Shyamali C Dharmage 1,2 MD, PhD*

*Equal Senior Authors

**Appendix S1-ADDITIONAL METHODS**

**Tasmanian Longitudinal Health Study**

Briefly, TAHS began in 1968, when 8583 children born in 1961 (7 years old) were recruited from all the schools in Tasmania, and it was followed up to 2012-2016 when participants were 53. Follow-ups of this cohort occurred in 1974, 1979-1981, 1992, 2004-2006, 2010, 2012-2016, and 2021-until now. During the 1968 survey, parents completed questionnaires on their children's respiratory health, while these participants completed self-administered questionnaires in subsequent follow-ups. Various health parameters were measured at each follow-up, including lung function (spirometry), anthropometrics, environmental exposures, and family histories of smoking and respiratory diseases. The current study includes TAHS participants (n=4194) whose weight and height were documented prospectively. However, data for LF were only available for half of these participants during middle age [1].

**BMI Trajectories**

Although BMI trajectories were developed and published previously, I would like to explain some methodological issues here. The current analysis included participants whose weights and heights were recorded from childhood to middle age. Weights and heights were measured at baseline and all follow-ups. Previously, their weights and heights were measured and recorded regularly in school medical records by school nurses, and these data were extracted. Thus, we had data available for analyses at ages 7, 13, 20, 31, and 43 years collected in the study follow-ups and at 5-6 years, 10-11 years, and 14-15 years from the school medical records, giving a total of 8-time points. BMI (weight /height^2^) was converted into age and sex-specific BMI z-scores based on the observed values within this cohort, as there were no standard equations for an Australian adult population.

The Low trajectory (26.7%) consistently exhibited the lowest BMI at all time points, starting below 15 kg/m² at 5 years (with a BMI z-score around -1), gradually rising to about 25 kg/m² (while maintaining a z-score around -1 across the lifespan). The Average trajectory (50.3%), the largest group, maintained steady BMI values between 16-25 kg/m² (with a stable z-score around zero), serving as the reference group. The Child high-decreasing trajectory (14.1%) started with a high BMI (and BMI z-score around 1.2), started around 17 kg/m² in early childhood before gradually declining to align with the average trajectory in adulthood, (ultimately reaching a z-score near zero). Although it keeps increasing but the rate of increase/velocity becomes slower than other (a decreasing z-score). The Child average-increasing trajectory (5.8%), increasing rapidly over time to reach approximately 37 kg/m² (with a BMI z-score around zero in early life and increasing to 2 in adulthood). Finally, the High trajectory (3.1%) started with the highest BMI (and z-scores), starting above 18 kg/m² (with a z-score over 2 at 5 years), rising sharply to approximately 35 kg/m² (with a z-score around 3 in adolescence), and then keeps increasing but at a slower rate (e.g. slightly decreasing z-score) during adulthood.We used two data sets to increase the BMI time points. Weights and heights in both data sources were measured by trained staff. School Health Nurses measured the weights and heights and recorded them in school medical records. The trained TAHS study staff measured the data in TAHS follow-ups. Furthermore, to address the potential methodological issue using different data sources, we assessed the correlation between the data collected by school nurses and TAHS-trained researchers at seven years of age. The correlation between the two data sources in a sample of 669 participants was substantial and statistically significant, rs: 0.75, p: <0.001". Given that the two sources of measurement for each participant were not measured simultaneously in the two data sets, less a perfect correlation between the two sets is expected. Similarly, the correlation between self-reported data at 43 years and measured data at 45 years (n-1360) was substantial (rs = .88, p = <0.001). Therefore, we believe that using two data sources at both time points did not influence our findings.

**Statistical Analysis**

The distributions of the early life risk factors of the BMI trajectories were assessed using the chi-square test for categorical variables and ANOVA for continuous variables if assumptions were fulfilled.

The minimum confounders included sex, birth weight, breastfeeding, mother's age, parental history of smoking, mother's education, family history of asthma, respiratory tract infections, physical activity, allergies, socioeconomic status, and number of siblings. We adjusted the association between BMI trajectories and adult lung function for the minimum variables, except birthweight, breastfeeding, physical activity, diet, and maternal education. However, we used 'feeding in the first three months' as a proxy for breastfeeding, small for gestational age as a proxy for birth weight and gestational age, maternal employment as a proxy for maternal education, and adult education and employment as a proxy for adulthood SES. Results are presented as odds ratios with 95% CI and a p-value =0.05, considered statistically significant.

**Description of other variables**

The information about these variables was collected contemporaneously by questionnaires. Childhood variables were defined using the information provided by parents in the 1968 survey when the children were seven years of age. Adulthood variables were collected from the 43-year follow-up.

**Childhood Variables**

Childhood asthma was measured as yes or no by asking the question: has he/she at any time in his/her life suffered from attacks of asthma or wheezy breathing? Childhood respiratory infections were measured by considering the questions for bronchitis, pneumonia, chest illness and tonsillitis. Childhood bronchitis was considered a binary variable, yes/no, using the question: has he/she at any time in life suffered from bronchitis or cough attacks with sputum in the chest? Childhood pneumonia was measured as yes or no with a question: have you ever been told by a doctor that they had pneumonia or pleurisy? Childhood chest illness was considered as not at all, 1-7 days, and more than 7 with a question: for how much time in the past twelve months has the child been confined to the house because of the chest illness? Tonsillectomy is measured as yes or no by asking if he/she has had the tonsils removed. Childhood food allergy was measured as yes or no using the question: has a doctor told you that he/she is allergic to any food or medicine? Childhood socioeconomic status was classified by the father's occupation and divided into five categories using the Australian Standard Classification of Occupation [2]. The mother's age was considered as a continuous variable. Parental smoking history was defined by the parent's responses to the question: do you smoke every day or six days out of seven?

Similarly, parental asthma was defined by the parent's response to the question; have you ever had asthma or an attack of wheezing like asthma? Data on birth weight and breastfeeding was available for a subset; therefore, to adjust for birthweight and gestational age, we developed a "small for gestational age" variable and adjusted for SGA in this subset [3]. Physical activity, diet and parental education were considered potential confounders but were not included due to insufficient information.

**Adulthood Variables**

We did not adjust for all the adulthood factors due to high collinearity with childhood risk factors. However, adulthood food allergy was measured as a binary variable by asking, have you ever been allergic to any food? Employment (are you currently employed or self-employed?) and education achievement (what is the highest educational or vocational qualification that you have completed) were used as proxy variables for adulthood socioeconomic status. Employment was considered as a binary variable (yes/no), and education was classified as less than to 12^th^ grade or more than and equal to 12 grades based on the due to sparse data in different categories. Adulthood smoking was measured as yes/no by asking: have you smoked at least 100 cigarettes or equal amounts of cigars, pipes, or any other tobacco product in your lifetime?

**Transfer factor for carbon monoxide of the lungs (T_L_co)**

Single breath nitrogen washout TL_CO_ values were performed according to the ATS/ERS guidelines being two technically acceptable TL_CO_ measurements within 10% or 3ml CO (STPD)/min/mm Hg of the average TL_CO_ [4], and included an adjustment to a standard hemoglobin concentration and correction for the presence carboxyhemoglobin. TL_CO_ values were converted to SI GLI values using GLI reference equations by Stanojevic et al [5]. GLI reference values were used to derive age, height, and gender predicted TL_CO_ and lower limit of TL_CO_.

**Lung static volume measurements**

Lung volume measurements were performed according to the ATS/ERS guidelines, where total lung capacity (TLC) was derived by adding functional residual capacity (FRC) to inspiratory capacity (IC), from the mean of two technically acceptable measurements (agreement within 5%) [6]. Expiratory reserve volume (ERV) was derived by subtracting residual volume (RV) from FRC.

**Figure S1. DAG for the Association of BMI Trajectories and COPD**


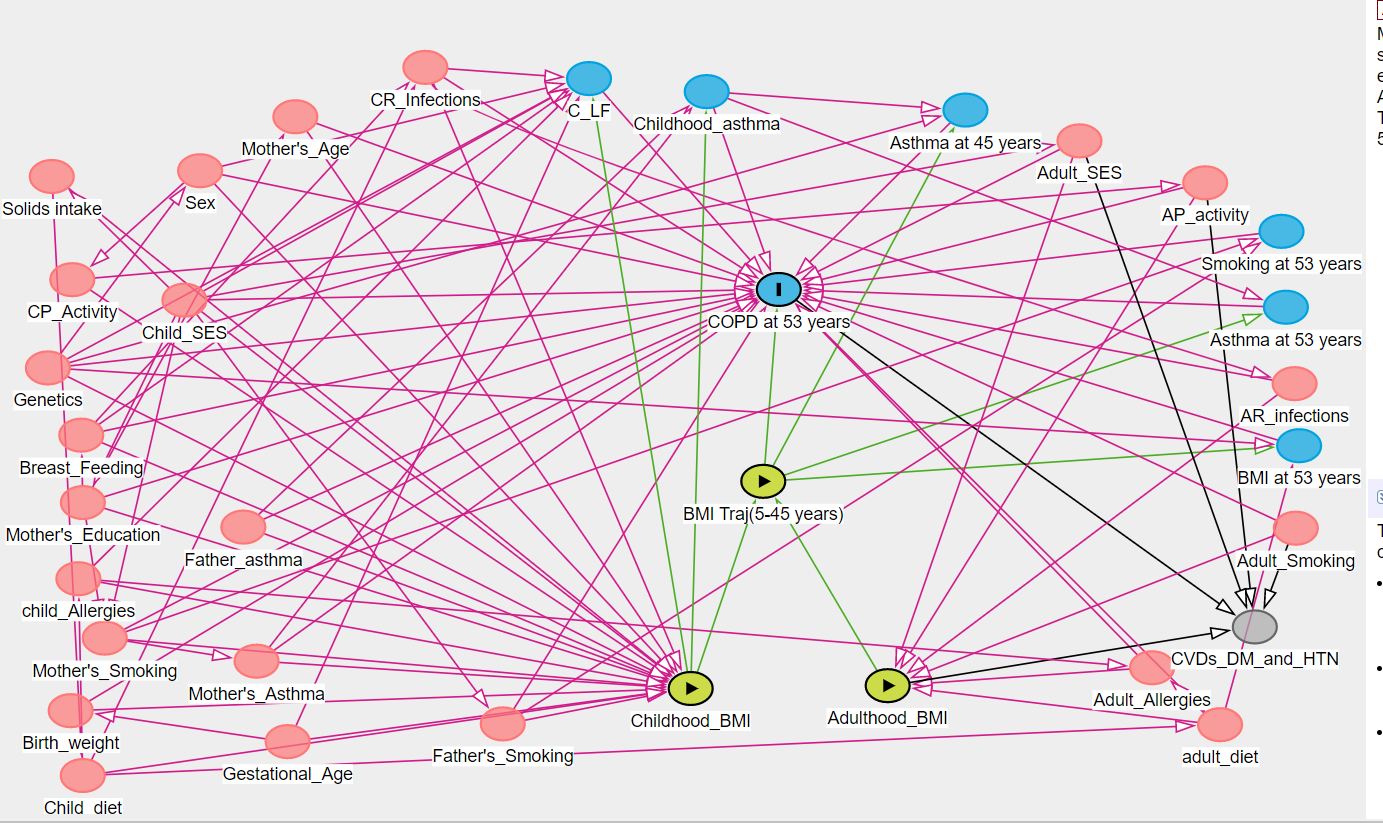


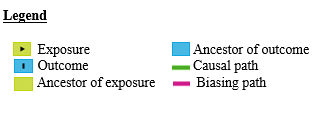


**Figure S2. DAG for the Association of BMI Trajectories and Lung Function**


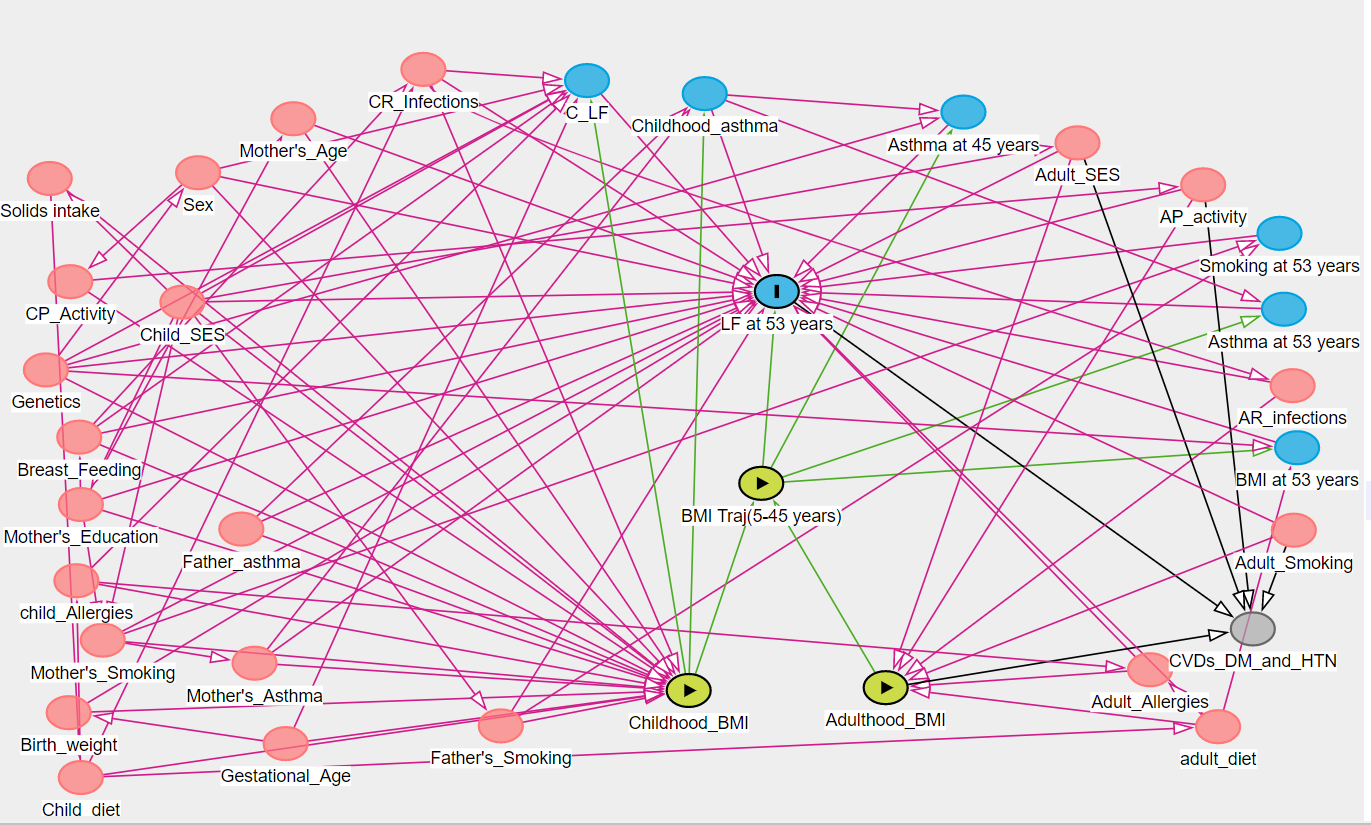


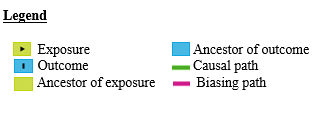


**Figure S3**

**Figure S4**

**Figure S5**

**Figure S6**

**Table S1. Association of BMI trajectories (5-43 years) with the Lung Function at the age of 45 years**

| Lung Function Indicators | BMI Trajectories | n | Adjusted Mean (SD) | Coefficient^†^ (95%CI), P-value |
| --- | --- | --- | --- | --- |
| Post-BD FEV_1_  (mL) | Average  Low  Child high-decreasing  Child average-increasing  High | 434  239  133  55  29 | 3299.6 (115.1)  3175.7 (118.6)  3313.7 (121.7)  3238.1 (129.5)  3156.8 (140.5) | n= 890  -  **-123.9 (-196.4, -51.4), 0.001**  14 (-75.5, 103.6), 0.75  -61.6 (-191.7, 68.5), 0.35  -142.8 (-316.2, 30.5), 0.11 |
| Post-BD FVC  (mL) | Average  Low  Child high-decreasing  Child average-increasing  High | 434  239  133  55  29 | 4409.8 (137.9)  4316.3 (142.2)  4384.6 (146.2)  4243.6 (154.1)  4232.7 (167.1) | n= 890  -  **-90.5 (-173.4, -7.7), 0.03**  -41.5 (-143.8, 60.9), 0.42  -65.8 (-214.5, 82.9), 0.38  -116.9 (-315.0, 81.2), 0.25 |
| Post-BD FEV_1_/FVC | Average  Low  Child high-decreasing  Child average-increasing  High | 434  239  133  55  29 | 74.9 (1.65)  73.8 (1.70)  75.9 (1.75)  74.8 (1.8)  73.9 (2.02) | n= 890  -  **-1.2 (-2.2, -0.1), 0.03**  0.9 (-0.3, 2.2), 0.15  -0.2 (-2.0, 1.7), 0.86  -1.1 (-3.6, 1.4), 0.39 |
| ^†^Sex, Type of Feeding in The First Three Months, No of Siblings, Chest Illness, Tonsillectomy, Pneumonia, Childhood Food Allergy, Bronchitis, Social Class During Childhood, Mother's Employment, Mother's Age, Mother's Asthma, Mother's Smoking, Father's Asthma, Father's Smoking, Adulthood Education, Adulthood Food Allergy Height at 45 years and age at 45 years | | | | |

**Table S2. Associations of BMI trajectories (5-43 years) with the Lung Function at the age of 53 years**

| Lung Function Indicators | BMI Trajectories | n | Adjusted Mean (SD) | Coefficient^†^ (95%CI), P-value |
| --- | --- | --- | --- | --- |
| Post-BD FEV_1_  (mL) | All Time Average  Low  Child high-decreasing  Child average-increasing  High | 816  465  239  83  48 | 3080.3 (100.0)  3013.9 (101.6)  3097.0 (102.5)  2982.7 (110.0)  3020.5 (118.1) | n=1651  -  **-66.5 (-119.7, -13.1), 0.015**  16.7 (-50.8, 84.2), 0.63  -97.6 (-203.6, 8.4), 0.07  -59.7 (-195.9, 76.5), 0.39 |
| Post-BD FVC  (mL) | Average  Low  Child high-decreasing  Child average-increasing  High | 816  465  239  83  48 | 4355.2 (111.4)  4289.9 (113.1)  4334.7 (114.1)  4128.1 (122.5)  4232.7 (131.5) | n=1651  -  **-65.4 (-124.7, -5.9), 0.03**  -20.5 (-95.7, 54.7), 0.59  **-227.2 (-345.3, -109.1), <0.001**  -122.5 (-274.3, 29.3), 0.11 |
| Post-BD FEV_1_/FVC | Average  Low  Child high-decreasing  Child average-increasing  High | 816  465  239  83  48 | 70.9 (1.45)  70.5 (1.46)  71.5 (1.48)  72.9 (1.59)  71.5 (1.71) | n=1651  -  -0.2 (-0.9, 0.5), 0.63  0.8 (-0.1, 1.7), 0.08  **1.6 (0.1, 2.9), 0.03**  0.8 (-1.2, 2.5)0.46 |
| ^†^ Sex, Type of Feeding in The First Three Months, No of Siblings, Chest Illness, Tonsillectomy, Pneumonia, Childhood Food Allergy, Bronchitis, Social Class During Childhood, Mother's Employment, Mother's Age, Mother's Asthma, Mother's Smoking, Father's Asthma, Father's Smoking, Adulthood Education, Adulthood Food Allergy height at 53 and age at 53 years | | | | |

**Table S3. Association of BMI trajectories (5-43 years) with the Lung Function decline from 45 to 53 years.**

| Lung Function Indicators | BMI Trajectories | n | Adjusted Mean (SD) | Coefficient^†^ (95%CI), P-value |
| --- | --- | --- | --- | --- |
| Post-BD FEV_1_  (mL) | Average  Low  Child high-decreasing  Child average-increasing  High | 294  163  86  29  17 | -275.4 (80.2)  -275.8 (83.1)  -245.1 (84.4)  -255.9 (95.1)  -216.0 (96.7) | n= 589  -  -0.5 (-48.6, 47.7), 0.98  30.2 (-30.4, 90.8), 0.32  -80.6 (-178.0, 16.9), 0.11  59.3 (-64.8, 183.4), 0.34 |
| Post-BD FVC  (mL) | Average  Low  Child high-decreasing  Child average-increasing  High | 294  163  86  29  17 | -31.9 (101.4)  -62.6 (104.9)  -45.8 (106.5)  -210.0 (119.9)  -49.5 (122.1) | n= 589  -  -30.6 (-91.2, 29.9), 0.32  -13.9 (-90.3, 62.6), 0.72  **-178.0 (-300.6, -55.4), 0.005**  -17.5 (-173.6, 138.6), 0.83 |
| Post-BD FEV_1_/FVC | Average  Low  Child high-decreasing  Child average-increasing  High | 294  163  86  29  17 | 72.9 (1.6)  73.4 (1.6)  73.7 (1.7)  72.3 (1.8)  73.8 (1.9) | n= 589  -  0.4 (-0.5, 1.3), 0.40  0.7 (-0.5, 1.9), 0.23  -0.7 (-2.6, 1.2), 0.48  0.9 (-1.6, 3.4), 0.48 |
| ^†^Sex, Type of Feeding in The First Three Month, No of Siblings, Chest Illness, Tonsillectomy, Pneumonia, Childhood Food Allergy, Bronchitis, Social Class During Childhood, Mother's Employment, Mother's Age, Mother's Asthma, Mother's Smoking, Father's Asthma, Father's Smoking, Adulthood Education, Adulthood Food Allergy, Current Employment, Baseline lung function, Change in Height 45-53 and Change in Age45-53 | | | | |

**Table S4. Associations of BMI trajectories (5-43 years) with the Obstructive and Spirometric restrictive Lung Function at the age of 45 years**

| Lung Function Indicators  BMI Trajectories | n | Normal lung Function  Base Outcome | n | Spirometric restrictive Only Adjusted MOR (95%CI), P-value | n | Obstructive Only  Adjusted MOR (95%CI), P-value |  |
| --- | --- | --- | --- | --- | --- | --- | --- |
| Average (n=517)  Low (n=280)  Child high-decreasing (n=155)  Child average-increasing (n=63)  High (n=35) | 462  238  145  57  26 | -  -  -  -  - | 16  10  2  1  4 | -  1.58 (0.62, 3.98), 0.34  0.58 (0.12, 2.76), 0.49  0.61(0.07, 5.20), 0.61  2.76 (0.51, 15.14), 0.24 | 39  32  8  5  5 | -  1.36 (0.76, 2.41), 0.30  0.57 (0.23, 1.43), 0.23  0.70 (0.20, 2.47), 0.59  1.16 (0.31, 4.38), 0.82 |  |
| * Sex, Type of Feeding in The First Three Months, No of Siblings, Chest Illness, Tonsillectomy, Pneumonia, Childhood Food Allergy, Bronchitis, Social Class During Childhood, Mother's Employment, Mother's Age, Mother's Asthma, Mother's Smoking, Father's Asthma, Father's Smoking, Adulthood Education, Adulthood Food Allergy height at 45 and age at 45 years | | | | | | | |

**Table S5. Association of BMI trajectories (5-43 years) with COPD at 45 years**

| BMI Trajectories | n/N | Crude  OR (95%CI), P-Value  (n=890) | Adjusted ⃰  OR (95%CI), P-Value  (n=890) |
| --- | --- | --- | --- |
| Average  Low  Child high-decreasing  Child average-increasing  High | 38/434  26/239  6/133  6/55  3/29 | -  1.27 (0.75, 2.15), 0.37  0.49 (0.20, 1.19), 0.12  0.82 (0.28, 2.38), 0.71  1.20 (0.34, 4.15), 0.77 | -  1.46 (0.84, 2.54), 0.18  0.54 (0.22, 1.37), 0.19  0.89 (0.29, 2.71), 0.83  1.03 (0.27, 3.82), 0.96 |
| * Sex, Type of Feeding in The First Three Months, No of Siblings, Chest Illness, Tonsillectomy, Pneumonia, Childhood Food Allergy, Bronchitis, Social Class During Childhood, Mother's Employment, Mother's Age, Mother's Asthma, Mother's Smoking, Father's Asthma, Father's Smoking, Adulthood Education, Adulthood Food Allergy height at 45 and age at 45 years | | | |

**Table S6. Associations of BMI trajectories (5-43 years) with the Obstructive and Spirometric restrictive Lung Function at the age of 53 years**

| Lung Function Indicators  BMI Trajectories | n | Normal lung Function  Base Outcome | n | Spirometric restrictive Only, Adjusted MOR (95%CI), P-value | n | Obstructive Only  Adjusted MOR (95%CI), P-value |
| --- | --- | --- | --- | --- | --- | --- |
| Average (n=935)  Low (n=528)  Child high-decreasing (n=266)  Child average-increasing (n=93)  High (n=53) | 877  479  252  90  44 | -  -  -  -  - | 14  14  3  2  4 | -  1.65 (0.74, 3.67), 0.22  0.79 (0.22, 2.85), 0.72  1.55 (0.33, 7.23), 0.56  **6.95 (2.04, 23.69), 0.002** | 44  35  11  1  5 | -  1.29 (0.76, 2.21), 0.30  0.95 (0.46, 1.96), 0.89  0.21 (0.03, 1.56), 0.13  1.39 (0.39, 4.95), 0.61 |
| * Sex, Type of Feeding in The First Three Months, No of Siblings, Chest Illness, Tonsillectomy, Pneumonia, Childhood Food Allergy, Bronchitis, Social Class During Childhood, Mother's Employment, Mother's Age, Mother's Asthma, Mother's Smoking, Father's Asthma, Father's Smoking, Adulthood Education, Adulthood Food Allergy height at 53 and age at 53 years | | | | | | |

**Table S7. Association of BMI trajectories (5-43 years) with COPD at 53 years**

| BMI Trajectories | n/N% (n) | Crude  OR (95%CI), P-Value  (n=1632) | Adjusted ⃰  OR (95%CI), P-Value  (n=1632) |
| --- | --- | --- | --- |
| Average  Low  Child high-decreasing  Child average-increasing  High | 40/806  27/462  11/235  1/81  3/48 | Reference  1.19 (0.72, 1.96), 0.50  0.94 (0.47, 1.86), 0.86  0.24 (0.03, 1.76), 0.16  1.27 (0.38, 4.29), 0.69 | Reference  1.32 (0.78, 2.22), 0.30  0.92 (0.45, 1.90), 0.83  0.19 (0.03, 1.48), 0.11  1.24 (0.35, 4.35), 0.74 |
| * Sex, Type of Feeding in The First Three Months, No of Siblings, Chest Illness, Tonsillectomy, Pneumonia, Childhood Food Allergy, Bronchitis, Social Class During Childhood, Mother's Employment, Mother's Age, Mother's Asthma, Mother's Smoking, Father's Asthma, Father's Smoking, Adulthood Education, Adulthood Food Allergy height at 53 and age at 53 years | | | |

**Table S8: P-values of the likelihood ratio test for the interaction of selected variables with BMI trajectories to predict the outcome as lung function**

| **Variables interacting with BMI trajectories** | **Lung Function Indices at 45 years**  **P-value for interaction** | **Lung Function Indices at 53 years**  **P-value for interaction** | **COPD at 45 years**  **P-value** | **COPD at 53 years**  **P-value** |
| --- | --- | --- | --- | --- |
| **Sex** | FEV_1_ - post-BD= 0.71  FVC - post-BD=0.27  FEV_1_/FVC - post-BD= 0.69 | FEV_1_ -post-BD= 0.27  FVC - post-BD= 0.15  FEV_1_/FVC - post-BD= 0.32 | 0.27 | 0.32 |
| **Current Smoking** | FEV_1_ - post-BD= 0.87  FVC - post-BD= 0.62  FEV_1_/FVC - post-BD=0.76 | FEV_1_ - post-BD= 0.34  FVC - post-BD= 0.12  FEV_1_/FVC - post-BD= 0.65 | 0.79 | 0.18 |
| **Childhood Asthma** | FEV_1_ - post-BD= 0.39  FVC - post-BD=0.13  FEV_1_/FVC - post-BD=0.48 | **FEV_1_ - post-BD=0.04**  **FVC- post-BD=0.07**  FEV_1_/FVC - post-BD=0.59 | 0.22 | 0.25 |
| **Current BMI** | FEV_1_ - post-BD= 0.68  FVC - post-BD= 0.42  FEV_1_/FVC - post-BD=0.17 | FEV_1_ - post-BD= 0.58  FVC - post-BD= 0.81  FEV_1_/FVC- post-BD= 0.30 | 0.14 | 0.54 |
| **Childhood Lung Function** | FEV_1_ - Pre-BD= 0.61  FVC- Pre-BD= 0.73  FEV_1_/FVC -Pre-BD= 0.33 | FEV_1_ - Pre-BD= 0.52  FVC- Pre-BD= 0.15  FEV_1_/FVC -Pre-BD= 0.36 | **-** | **-** |

**Table S9. Associations^*^ of BMI trajectories (5-43 years) with the Lung Function at the age of 53 years, stratified by Childhood Asthma**

| Lung Function Indicators | BMI Trajectories | n | Adjusted Mean (SD) | Coefficient (95%CI), P-value  Without Asthma | n | Adjusted Mean (SD) | Coefficient (95%CI), P-value  With Asthma | P-Interaction Term |
| --- | --- | --- | --- | --- | --- | --- | --- | --- |
| ^§^Post-BD FEV_1_  (mL) | Average  Low  Child high-decreasing  Child average-increasing  High | 674  398  198  68  40 | 3092.8 (128.3)  3043.6 (128.8)  3108.9 (129.9)  3011.9 (136.2)  2961.2 (146.4) | n=1378  -  -49.1 (-106.8, 8.5), 0.09  16.1 (-57.6, 89.8), 0.66  -80.8 (-197.3, 35.6), 0.17  -131.6 (-279.8, 16.7), 0.08 | 142  67  41  15  8 | 3350.1 (225.9)  3186.9 (242.2)  3346.3 (240.7)  3251.5 (272.1)  3621.9 (276.8) | n=273  -  **-163.2 (-309.2, -17.2), 0.02**  -3.8 (-176.8, 169.3), 0.96  -98.6 (-371.3, 174.1), 0.47  271.8 (-80.2, 623.8), 0.12 | **0.07**  0.74  0.50  0.03 |
| ^§^Post-BD  FVC  (mL) | Average  Low  Child high-decreasing  Child average-increasing  High | 674  398  198  68  40 | 4370.7 (142.8)  4328.8 (143.4)  4355.1 (144.7)  4153.5 (151.6)  4188.7 (162.9) | n=1378  -  -41.9 (-106.1, 22.3), 0.20  -15.6 (-97.7, 66.4), 0.71  **-217.2 (-346.9, -87.6), 0.001**  **-181.9 (-347.0, -16.9), 0.03** | 142  67  41  15  8 | 4517.8 (249.7)  4305.6 (267.7)  4439.5 (266.1)  4253.4 (300.8)  4731.8 (306.1) | n=273  -  **-212.3 (-373.6, -50.9), 0.01**  -78.4 (-269.6, 112.9), 0.42  -264.5 (-565.9, 36.9), 0.08  213.9 (-175.2, 602.9), 0.28 | **0.04**  0.95  0.52  0.10 |
| * Sex, Type of Feeding in The First Three Months, No of Siblings, Chest Illness, Tonsillectomy, Pneumonia, Childhood Food Allergy, Bronchitis, Social Class During Childhood, Mother's Employment, Mother's Age, Mother's Asthma, Mother's Smoking, Father's Asthma, Father's Smoking, Adulthood Education, Adulthood Food Allergy height at 53 and age at 53 years.  § P-value for Interaction <0.1 | | | | | | | | |

**Table S10. Association of BMI trajectories (5-43 years) with the TL_CO_ and Static Lung Volumes at 45 years in a subset of participants**

| **Lung Function Indicators** |  | | **Average** | **Low** | **Child high-decreasing** | **Child average-increasing** | **High** |
| --- | --- | --- | --- | --- | --- | --- | --- |
| **TL_CO_ (**ml/min/mm Hg) | **n** | | 391 | 211 | 118 | 40 | 25 |
|  | **M(SD)** | | 27.3 (6.8) | 26.0 (6.7) | 28.1 (7.2) | 27.8 (6.3) | 27.9 (7.7) |
|  | **Coefficient** |  | - | -0.2 (-0.4, 0.04), 0.1 | 0.3 (-0.1, 0.6), 0.09 | **0.6 (0.2, 1.1), 0.01** | **1.01 (0.4, 1.6), 0.001** |
| **Static Lung Volumes** |  |  |  |  |  |  |  |
|  | **n** | | 399 | 214 | 117 | 44 | 26 |
| **TLC (L)** | **M(SD)** | | 6.6 (1.3) | 6.4 (1.3) | 6.5 (1.3) | 6.2 (1.2) | 5.9 (0.9) |
|  | **Coefficient (95%CI), P-value** | | - | -0.1 (-0.2, 0.04), 0.2 | 0.01 (-0.1, 0.2), 0.8 | -0.01 (-0.2, 0.2), 0.8 | -0.3 (-0.6, 0.04), 0.09 |
| **FRC (L)** | **M(SD)** | | 3.2 (0.7) | 3.2 (0.8) | 3.1 (0.7) | 2.6 (0.6) | 2.5 (0.6) |
|  | **Coefficient (95%CI), P-value** | | - | 0.1 (-0.02, 0.2), 0.1 | -0.04 (-0.2, 0.1), 0.5 | **-0.3 (-0.5, -0.1), 0.003** | **-0.4 (-0.6, -0.1), 0.003** |
| **ERV (L)** | **M(SD)** | | 1.2 (0.5) | 1.3 (0.5) | 1.1 (0.4) | 0.8 (0.4) | 0.8 (0.5) |
|  | **Coefficient (95%CI), P-value** | | - | **0.1 (0.03, 0.2), 0.01** | -0.05 (-0.1, 0.1), 0.3 | **-0.3 (-0.4, -0.2), <0.001** | **-0.2 (-0.4, -0.1), 0.01** |
| **RV (L)** | **M(SD)** | | 1.9 (0.5) | 1.9 (0.6 | 1.9 (0.5) | 1.8 (0.4) | 1.7 (0.4) |
|  | **Coefficient (95%CI), P-value** | | - | -0.02 (-0.1, 0.1), 0.7 | -0.001 (-0.1, 0.1), 0.9 | -0.02 (-0.1, 0.2), 0.8 | -0.2 (-0.3, 0.1), 0.2 |
| Variables Adjusted: Sex, Type of Feeding in The First Three Months, Chest Illness, Tonsillectomy, Pneumonia, Childhood Food Allergy, Bronchitis, Social Class During Childhood, Mother's Employment, Mother's Age, Mother's Asthma, Mother's Smoking, Father's Asthma, Father's Smoking, Adulthood Education, Adulthood Food Allergy Height at 45 years and age at 45 years | | | | | | | |

**Table S11. Association of BMI trajectories (5-43 years) with the TL_CO_ and IRV at 53 years in a subset of participants**

| Lung Function Indicators | TL_CO_ (ml/min/mm Hg) | | | IRV (L) | | |
| --- | --- | --- | --- | --- | --- | --- |
| Lung Function Indicators | **n** | **M (SD)** | **Coefficient**  **(95%CI), P-value** | **n** | **M(SD)** | **Coefficient**  **(95%CI), P-value** |
| Average  Low  Child high-decreasing  Child average-increasing  High | 790  444  237  78  48 | 24.9 (7.2)  24.7 (6.7)  26.2 (7.1)  24.9 (6.8)  24.9 (4.7) | Reference  -0.2 (-0.8, 0.4), 0.4  1.11 (-0.1, 2.3), 0.08  **1.2 (0.4, 1.9), 0.002**  **1.72 (0.2, 3.2), 0.03** | 796  449  239  80  48 | 4.2 (0.1)  4.1 (0.1)  4.2 (0.2)  4.0 (0.2)  4.2 (0.2) | Reference  -0.04 (-0.1, 0.1), 0.4  0.03 (-0.1, 0.1), 0.6  -0.1 (-0.3, 0.1), 0.2  0.002 (-0.2, 0.2), 0.9 |
| Variables Adjusted: Sex, Type of Feeding in The First Three Months, Chest Illness, Tonsillectomy, Pneumonia, Childhood Food Allergy, Bronchitis, Social Class During Childhood, Mother's Employment, Mother's Age, Mother's Asthma, Mother's Smoking, Father's Asthma, Father's Smoking, Adulthood Education, Adulthood Food Allergy, Height at 53 years and age at 53 years | | | | | | |

**Table S12 Characteristics of participants lost to follow-up from 43 to 53 years compared to those who remained in the study.**

| Characteristics | With Lung function data at 53 among those with BMI trajectory data  N=2482 | Without Lung function data at 53 among those with BMI trajectory data  N=1712 | P-Value |
| --- | --- | --- | --- |
| BMI Trajectory Exposure 5-43 years |  |  |  |
| BMI Trajectories – % (n)  Average  Low  Child High-Decreasing  Child Average-Increasing  High | 49.9 (962)  27.9 (539)  14.4 (277)  5.1 (98)  2.8 (53) | 50.6 (1147)  25.7 (582)  13.9 (315)  6.5 (147)  3.3 (75) | 0.15 |
|  |  |  |  |
| FEV_1_ at 7 years-mL-m (sd) | 1347.2 (214.1) | 1335.3 (215.7) | 0.09 |
| FVC at 7 years- mL-m (sd) | 1474.5 (242.5) | 1.460.1 (243.4) | 0.06 |
| FEV_1_/FVC at 7 years- mL-m (sd) | 0.91 (0.06) | 0.91 (0.06) | 0.83 |
| Childhood Asthma– % (n) | 17.7 (337) | 16.7 (374) | 0.39 |
| Childhood Bronchitis– % (n) | 50.3 (957) | 48.9 (1094) | 0.37 |
| Childhood No of Chest illness – % (n)  Not at All  1-7 Days  More than 7 Days | 63.2 (1178)  27.6 (514)  9.3 (173) | 65.4 (1439)  25.8 (567)  8.8 (193) | 0.32 |

**Table S13: Characteristics of the sub-sample selected for the BMI trajectory development compared to the remaining in the original cohort**

| Characteristics | Selected Sub-sample in this study  n=4194 | Remaining participants in the Cohort  n=4390 |
| --- | --- | --- |
| Childhood Food Allergy – n (%) | 305 (7.4) | 265 (6.4) |
| Childhood Asthma – n (%) | 711 (17.2) | 688 (16.4) |
| Mother's asthma – n (%) | 441 (11.0) | 435 (10.9) |
| Father's asthma – n (%) | 417 (10.6) | 421 (10.9) |
| FEV_1_ at seven years – mean (SD) | 1340 (215.4) | 1319 (233.5) |
| FVC at 7 years – mean (SD) | 1466 (243.2) | 1443 (264.7) |
| FEV_1_/FVC at 7 Years – mean (SD) | 0.91 (0.06) | 0.91 (0.06) |

**References**

1. Matheson MC, Abramson MJ, Allen K, Benke G, Burgess JA, Dowty JG, et al. Cohort profile: the Tasmanian longitudinal health study (TAHS). Int J Epidemiol. 2017;46(2):407-8i.

2. Australian Bureau of Statistics. Australian standard classification of occupations.: ABS Canberra; 1997.

3. Roberts CL, Lancaster PA. Australian national birthweight percentiles by gestational age. Medical Journal of Australia. 1999;170(3):114-8.

4. Macintyre N, Crapo R, Viegi G, Johnson D, Van der Grinten C, Brusasco V, et al. Standardisation of the single-breath determination of carbon monoxide uptake in the lung. European Respiratory Journal. 2005;26(4):720-35.

5. Stanojevic S, Graham BL, Cooper BG, Thompson BR, Carter KW, Francis RW, et al. Official ERS technical standards: Global Lung Function Initiative reference values for the carbon monoxide transfer factor for Caucasians. European Respiratory Journal. 2017;50(3).

6. Quanjer PH, Tammeling G, Cotes J, Pedersen O, Peslin R, Yernault J. Lung volumes and forced ventilatory flows. European Respiratory Journal. 1993;6(Suppl 16):5-40.
